# Infection of hamsters with the UK *Clostridium* *difficile* ribotype 027 outbreak strain R20291

**DOI:** 10.1099/jmm.0.028514-0

**Published:** 2011-08

**Authors:** Anthony M. Buckley, Janice Spencer, Denise Candlish, June J. Irvine, Gillian R. Douce

**Affiliations:** Institute of Immunology, Infection and Inflammation, Glasgow Biomedical Research Centre, University of Glasgow, Glasgow G12 8QQ, UK

## Abstract

*Clostridium difficile* is the main cause of antibiotic-associated disease, a disease of high socio-economical importance that has recently been compounded by the global spread of the 027 (BI/NAP1/027) ribotype. *C*. *difficile* cases attributed to ribotype 027 strains have high recurrence rates (up to 36 %) and increased disease severity. The hamster model of infection is widely accepted as an appropriate model for studying aspects of *C*. *difficile* host–pathogen interactions. Using this model we characterized the infection kinetics of the UK 2006 outbreak strain, R20291. Hamsters were orally given a dose of clindamycin, followed 5 days later with 10 000 *C*. *difficile* spores. All 100 % of the hamsters succumbed to infection with a mean time to the clinical end point of 46.7 h. Colonization of the caecum and colon were observed 12 h post-infection reaching a maximum of approximately 3×10^4^ c.f.u. per organ, but spores were not detected until 24 h post-infection. At 36 h post-infection *C*. *difficile* numbers increased significantly to approximately 6×10^7^ c.f.u. per organ where numbers remained high until the clinical end point. Increasing levels of *in vivo* toxin production coincided with increases in *C*. *difficile* numbers in organs reaching a maximum at 36 h post-infection in the caecum. Epithelial destruction and polymorphonuclear leukocyte (PMN) recruitment occurred early on during infection (24 h) accumulating as gross microvilli damage, luminal PMN influx, and blood associated with mucosal muscle and microvilli. These data describe the fatal infection kinetics of the clinical UK epidemic *C*. *difficile* strain R20291 in the hamster infection model.

## Introduction

The Gram-positive, spore-forming bacillus *Clostridium difficile* is a pathogen of nosocomial importance causing a range of symptoms from asymptomatic carriage to severe diarrhoea, pseudomembranous colitis and death. *C*. *difficile* infection (CDI) typically occurs following antibiotic therapy, where disruption of the gut flora leaves the intestine susceptible to *C*. *difficile* colonization. Risk factors include the use of proton-pump inhibitors, antiperistaltic drugs, chemotherapy or immunosuppressive agents and old age (>65 years) (Cartman *et al.*, 2010).

In the last decade global incidence of CDI has increased dramatically, in the most part due to the emergence and spread of epidemic strains that are predominantly ribotype 027. Ribotype 027 strains are associated with more severe disease, increased mortality, higher relapse rates and increased resistance to fluoroquinolone antibiotics (Stabler *et al.*, 2009). In 2003 the first outbreak of ‘hypervirulent’ *C*. *difficile* 027 in the UK was reported, followed by another outbreak in 2005 (HC, 2006). During these two outbreaks, a total of 498 cases were reported with 172 deaths (HC, 2006). Since this first incursion several outbreaks due to ribotype 027 have been documented in almost all UK health board regions.

In 2007 there were 57 255 laboratory-confirmed cases in the UK (55.3 % were attributable to ribotype 027), but these numbers have steadily decreased to 28 458 cases in 2009 (36.1 % attributable to ribotype 027) [http://www.hpa.org.uk at Topics→Infectious Diseases→Infections A–Z→*Clostridium difficile*→Epidemiological Data *Clostridium difficile*→Voluntary Surveillance of *Clostridium difficile*→Results of the Voluntary Reporting (http://www.hpa.org.uk/web/HPAweb&Page&HPAwebAutoListName/Page/1179745282413)]. The total identifiable cost of CDI was estimated to be £4000 per case in England in 1996 (Wilcox *et al.*, 1996). On this basis such infections cost the UK £113 million in 2009 and £229 million in 2007.

The golden Syrian hamster is regarded as an important model of *C*. *difficile* disease as many of the clinical symptoms in humans can be observed in this model. After clindamycin administration and challenge with toxigenic *C. difficile* strains, hamsters develop a haemorrhagic caecitis, which manifests as ‘wet tail’ (a sign of diarrhoea in hamsters) and is followed by death (Bartlett *et al.*, 1977; Sambol *et al.*, 2001; Razaq *et al.*, 2007). Using this model we characterized the phenotypic outcome of infection with the clinical *C*. *difficile* ribotype 027 strain R20291. This strain was originally isolated from a patient who presented with pseudomembranous colitis from the first UK outbreak in 2006 and is considered an archetypal 027 strain within the UK.

## Methods

### 

#### Bacterial strain and spore preparation.

*C*. *difficile* strain R20291 was obtained from Professor Brendan Wren (London School of Hygiene and Tropical Medicine, London, UK) and is classed as ribotype 027 and toxinotype III (Rupnik *et al.*, 1998). Bacteria were grown on CCEY agar supplemented with cefoxitin–cycloserine, egg yolk emulsion (LabM) and erythromycin (100 µg ml^−1^) at 37 °C under anaerobic conditions. Spores were made according to Lawley *et al.* (2009) except strains were grown in brain heart infusion broth. Serial 10-fold dilutions of the spore preparation were inoculated onto supplemented CCEY agar plates to determine the number of c.f.u. ml^−1^, from which the inocula dilution was calculated.

#### Animal experiments.

Female golden Syrian hamsters that weighed approximately 100 g were purchased from Harlan Olac, UK. These animals were housed individually, and given water and food *ad libitum*. Telemetry chips (VitalView Emitter) were inserted by laparotomy into the body cavities of the animals at least 3 weeks before infection with *C*. *difficile*. Once the wounds healed, the animals were placed on receiver pads, and their body temperature and activity were monitored (VitalView software). Each animal received orogastrically 30 mg clindamycin phosphate kg^−1^ 5 days before infection. Following administration of the antibiotic, all animals were placed in individual sterilized isolator cages with disposable filter top lids, and were provided with sterile food, water and bedding. Each hamster received approximately 10 000 spores of strain R20291 (determined by serial dilutions before and after challenge, as described above). Experiments using 1, 3, 5 or 10 days post-clindamycin treatment and infection of hamsters with 100–100 000 spores have been tested previously; however, only the combination described above resulted in 100 % infection of the animals. Following infection, each animal was placed in a second sterile cage with appropriate food, water and bedding. At 12 (*n* = 6), 24 (*n* = 6) and 36 h post-challenge (*n* = 6), and when animals succumbed to infection (*n* = 13), animals were culled for post-mortem analysis. At each time point, to establish the level of colonization, the caecum and colon were removed aseptically. To enumerate the total bacterial load (spores and vegetative cells), each section was opened longitudinally, and the contents were removed by gentle washing in 10 ml PBS (luminal-associated bacteria). The tissues were washed in 10 ml PBS and homogenized in 5 ml PBS for 1 min using a stomacher (tissue-associated bacteria), and viable counts were determined for the homogenates. Serial 10-fold dilutions were plated on supplemented CCEY agar plates. To determine the numbers of spores present in the samples, the samples were heated for 10 min at 56 °C, and the numbers of spores present were determined by the viable count method as described above. Results are given as the mean number of recovered bacteria in at least six animals per time point.

#### Multilocus variable-number tandem-repeat analysis (MLVA).

To confirm the *C*. *difficile* isolated at post-mortem were the same strain originally used for infection, bacteria were isolated as single colonies, grown and the DNA isolated. PCR amplification of seven *C*. *difficile* repeat loci was performed using the primers and PCR amplification protocol described by Marsh *et al.* (2006). Following agarose gel electrophoresis MLVA patterns from spore inocula and recovered isolates were compared.

#### Detection of *in vivo* toxin levels.

Production of toxins by R20291 was detected *in vitro* using filtered caecum and colon content from animals that was taken during the post-mortem. Confluent monolayers of Vero cells (kidney epithelial cells) were maintained in Eagle’s minimal essential medium (EMEM; Sigma) supplemented with 1 % heat-inactivated fetal calf serum (Invitrogen), 2 mM l-glutamine (Invitrogen) and 100 µg penicillin/streptomycin ml^−1^. Cells were washed with preheated sterile PBS before addition of serial diluted filtered gut content in supplemented EMEM and incubated for 18 h at 37 °C (5 % CO_2_). Cells were washed with PBS, fixed in 1 % formalin for 10 min then washed again. Fixed adherent cells were stained with Giemsa for 30 min then washed before addition of 0.1 % SDS and left for 1 h. Optical density was measured using an EL_x_808 Ultra microplate reader (Bio-Tek Instruments) at 600 nm and compared to non-infected hamster caecal and colon gut contents as a negative control. If the toxin dilution is able to cause cell toxicity (cell rounding) this leads to the loss of cell adherence resulting in a reduced staining and hence optical density of the contents of the wells.

#### Histology.

Samples from different regions of the gut were prepared for simple histology as described by Goulding *et al.* (2009).

#### Scanning electron microscopy (SEM).

SEM samples were prepared as described by Goulding *et al.* (2009).

#### Statistical analysis.

All statistical analyses were performed using the GraphPad Prism 5 (GraphPad Prism Software). A Student’s one-tailed *t*-test was used to determine significant difference in bacterial recoveries between all time points examined. *P* values ≤0.05 were considered significant.

## Results

### Telemetry monitoring of infected hamsters

**Fig. 1.**
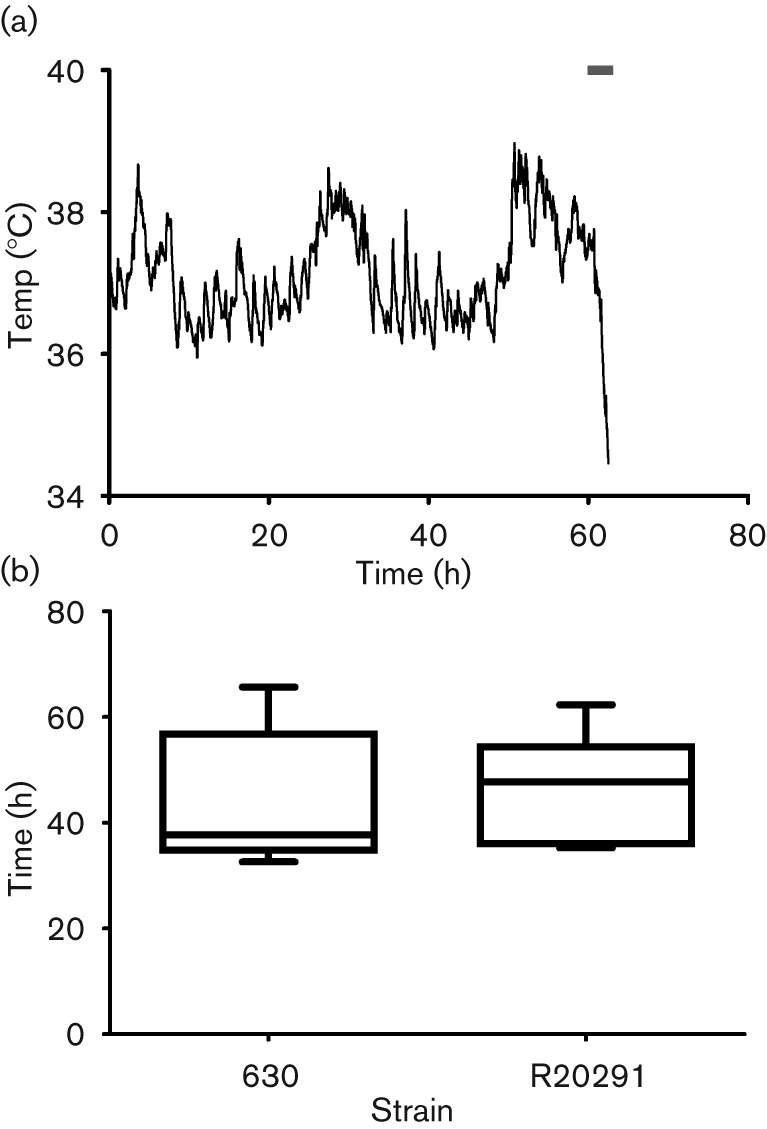
(a) Typical body temperature kinetics of a hamster infected with R20291. Hamsters were culled when body temperature declined below 35 °C (the clinical end point). The bar indicates when symptoms, typically ‘wet-tail’, manifested. (b) Box and whisker plot of time taken for animals to succumb to infection for strains 630 [data calculated from Goulding *et al.* (2009)] and R20291. The bottom and top of the box are the lower and upper quartile with the median time to clinical end point in the middle. The ends of the whiskers are the minimum and maximum times to the clinical end point.

If infection was allowed to progress to the clinical end point then the infection of hamsters with R20291 resulted in 100 % mortality. Hamsters typically followed the temperature profile shown in Fig. 1(a), where a rapid decrease in body temperature was observed following onset of clinical signs that included a wet tail (diarrhoea; represented by the bar in Fig. 1a). The drop in body temperature from 37 °C to the clinical end point (35 °C) took a mean time of 76±6.4 min sem. In comparison hamsters infected with *C*. *difficile* strain 630 displayed a significantly longer body temperature drop time (145±28.9 min; *P* = 0.02). The mean time taken for hamsters to succumb to infection was 46.7±2.8 h sem; however, a range between 35.2 and 62.3 h was observed (*n* = 13) (Fig. 1b). No significant difference was seen compared to hamsters infected with *C*. *difficile* strain 630 (43.7±3.7 h sem; *n* = 11).

### Colonization kinetics of R20291

**Fig. 2.**
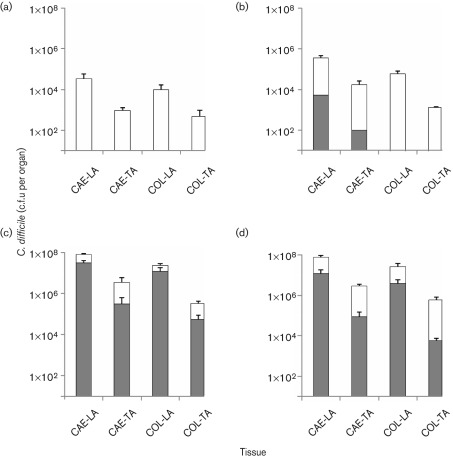
Colonization kinetics of *C*. *difficile* R20291 in hamsters. To monitor colonization throughout the infection process hamsters were culled at 12 (a), 24 (b) and 36 h (c) post-infection, and when the hamsters succumbed to infection (d). *C*. *difficile* was recovered from the caecum (CAE) and the colon (COL) associated with either the lumen (LA) or the tissue (TA). White bars represent total bacteria whilst grey bars indicate bacteria in spore form. Bacterial recoveries represent the geometric mean±sem (standard error of the mean) of at least two biological replicates, where each experiment used six animals per time interval.

To assess the colonization kinetics of *C*. *difficile* R20291 after inoculation, hamsters were culled at 12, 24 and 36 h post-challenge and at the clinical end point to measure the levels of R20291 in the caecum and colon. At 12 h after infection with 10^4^ spores per animal, *C*. *difficile* caecum levels reached approximately 3×10^4^ c.f.u. per organ with higher bacterial levels associated with the lumen compared to those associated with the tissue (Fig. 2a). Levels in the colon were lower than in the caecum, reaching approximately 1×10^4^ c.f.u. per organ. No *C*. *difficile* spores were detected in either the lumen or the tissue at this time. At 24 h post-challenge *C*. *difficile* levels increased in the caecum and colon (approximately 3×10^5^ and 6×10^4^ c.f.u. per organ, respectively) compared to 12 h, where more bacteria were associated with the lumen than tissue (*P* = 0.03 caecum only; Fig. 2b). *C*. *difficile* spores were present in low levels in the caecum (approximately 5×10^3^ c.f.u. per organ) but could not be detected in the colon. The rate of growth increased exponentially over the next 12 h, whereby at 36 h post-challenge mean caecal and colon levels were at approximately 5×10^7^, 3×10^6^, 1×10^7^ and 3×10^5^ c.f.u. per organ for caecum luminal associated (CAE-LA), caecum tissue associated (CAE-TA), colon luminal associated (COL-LA) and colon tissue associated (COL-TA) samples, respectively (Fig. 2c). Over the same 12 h period a significant increase in spores was observed at all sites sampled, with the highest levels observed at 36 h post-challenge in the lumen [60 (CAE-LA), 10 (CAE-TA), 80 (COL-LA) and 20 % (COL-TA) of total bacteria isolated, *P* = 0.01, 0.03, 0.04 and 0.06, respectively]. At the experimental end point (~46.7 h) the number of bacteria isolated from each site remained high and comparable to those observed at 36 h post-challenge (approximately 6×10^7^, 3×10^6^, 2×10^7^ and 6×10^5^ c.f.u. per organ for CAE-LA, CAE-TA, COL-LA and COL-TA samples, respectively; Fig. 2d). Compared to 36 h no significant differences in spore levels were seen at the clinical end point.

### MLVA

MLVA patterns for *C*. *difficile* isolated at the post-mortem analysis matched those MLVA patterns for the inoculating bacterial spores (data not shown).

### *In vivo* toxin levels

*In vivo* toxin production was measured semiquantitatively *in vitro* using a Vero cell toxicity assay by serially diluting filtered caecal and colon luminal contents, and calculating the maximum fold-dilution at which cell toxicity was still detected (cell rounding). Caecal and colon contents from animals culled at 12 and 24 h showed similar levels of toxin, approximate maximum 10- and 5-fold dilution required for cell rounding (caecum and colon samples, respectively; Table 1). In contrast, luminal contents taken at 36 h and at the clinical end point showed increased toxin abundance, approximate maximum 100 000- and 625-fold dilution required for cell rounding (caecum and colon samples, respectively; Table 1).

**Table 1.** Maximum fold dilution at which cell toxicity was still detected

Time post-challenge (h)	Fold dilution required for cell rounding	
	Caecum	Colon
12	Neat	Neat
24	10-fold	5-fold
36	10 000-fold	125-fold
Clinical end point	100 000-fold	625-fold

### Histological changes

**Fig. 3.**
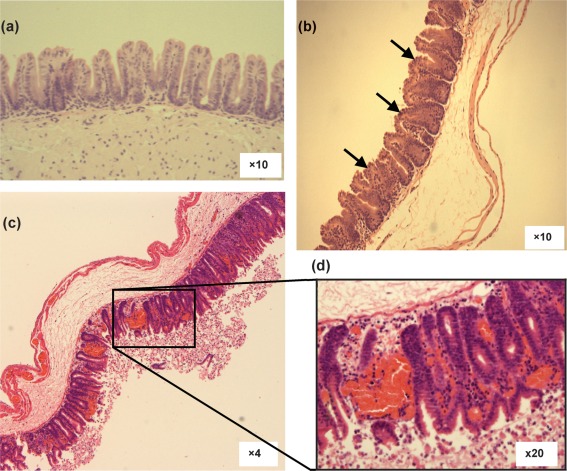
Caecal histology of hamsters infected with R20291. Samples were taken from uninfected controls (a), at 24 h post-infection (b) and at the clinical end point [(c) and inset (d)]. Arrows denote epithelial brush broader damage.

Gut pathology at 12, 24 and 36 h post-infection, and at the clinical end point was assessed by staining sections of gut tissue with haematoxylin and eosin, and comparing to uninfected controls. At 12 h post-infection, caecal and colon samples showed no change from uninfected controls (data not shown). By 24 h after infection an increase in polymorphonuclear leukocytes (PMNs) in the submucosa and microvilli of the caecum was seen along with the destruction of the greater part of the epithelial brush border exposing the circulating PMNs (Fig. 3b, indicated by arrows). Caecal and colon (data not shown) histology samples at 36 h and at the clinical end point showed severe destruction of the epithelial layer and extensive PMN influx in the lumen through ‘volcanic-like’ eruptions on the microvilli (Fig. 3c, d). A high association of red blood cells with the mucosal muscle and amongst the microvilli was observed, which is an indication of rapid and extensive damage presumably caused by the *C*. *difficile* toxins.

### SEM

**Fig. 4.**
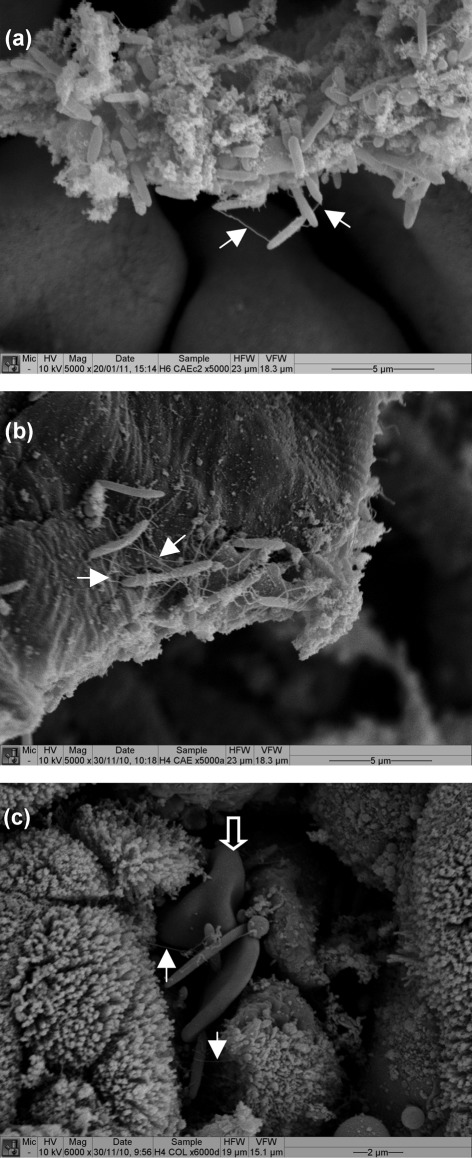
SEM of hamsters infected with R20291. Samples were taken from the caecum at 24 h post-infection (a), and from the caecum (b) and colon (c) at 36 h post-infection. Bacteria appeared to be interacting with the tissue surface and other bacteria via flagella-like appendages (filled arrows). In agreement with histology observations red blood cells were also observed in the same vicinity as bacteria (open arrow).

The detailed characterization of host–bacterial interactions was confirmed by SEM on caecum and colon samples taken at 12, 24 and 36 h post-infection and at the time of culling. At 12 h post-infection individual *Clostridium*-like bacterial cells could be seen sporadically on caecal surfaces. By 24 h post-infection a greater number of *Clostridium*-like bacteria were seen associated with mucus over both the tissue surface and crypt crevasses (Fig. 4a). During the height of bacterial recovery from the caecum and colon (36 h post-infection), aggregates of *Clostridium*-like bacteria could be seen on the tissue surface forming microcolony plaques typically associated over the crypt crevasses (Fig. 4b). Intimate tissue–bacterial and bacterial–bacterial interactions could be seen via flagella-like appendages; however, these structures could potentially be host derived (Fig. 4, indicated by filled arrows). In agreement with histology observations red blood cells were also observed in the same vicinity as *Clostridium*-like bacteria (Fig. 4c, indicated by open arrow).

## Discussion

Here we present a detailed study of the colonization kinetics of the UK *C. difficile* outbreak strain R20291 in hamsters. After infection with R20291 100 % of the animals succumbed to the infection, although the times to the clinical end point were spread over 20 h. The morbidity and mortality we observed are consistent with those seen with infection with high virulence strains in humans (Warny *et al.*, 2005). The diversity in times to the clinical end point could reflect the sensitivity of R20291 to the clindamycin used in the hamster model (MIC = 16 µg ml^−1^). These results are similar to those observed by Razaq *et al.* (2007) where infection with a clindamycin-resistant 027 ribotype isolate killed hamsters more quickly (40 h) compared to clindamycin-susceptible isolates (48 and 69 h). However, the mean time to the clinical end point seen with R20291 is very similar to ribotype 027 strains isolated in North America (Razaq *et al.*, 2007). Observed clinical-end points for *C*. *difficile* strain 630 (ribotype 012) were very similar to R20291; however, R20291 displayed a quicker temperature drop time compared to strain 630. This rapid temperature drop could be an indicator of enhanced virulence of these strains, an observation shared by Razaq *et al.* (2007), where strain 630 displayed a significantly longer time from *C*. *difficile* colonization to death compared to clindamycin-resistant 027 isolates.

Caecal and colon contents at 12 and 24 h post-infection showed lower recovery of *C*. *difficile* compared to clindamycin-resistant isolates, such as *C*. *difficile* M68 (data not shown), which suggests antibiotic killing of *C*. *difficile* by residual clindamycin levels. Larson & Borriello (1990) found that after a single 3 mg clindamycin dose *C*. *difficile* could still be detected in the caecum of hamsters after 11 days, which was enough to inhibit the growth of a clindamycin-susceptible strain. In this study treatment of hamsters with clindamycin was optimized to 5 days prior to challenge to give 100 % infection (resulting in 100 % mortality). Once gut colonization was established (24 h post-infection) rapid increases in bacterial numbers over a 12 h time period were observed (>2 log increase). This was quickly followed by morbidity, loss of body temperature and death (mean time to the clinical end point was 46.7 h). Razaq *et al.* (2007) reported mean times of colonization to death of between 7 and 16 h with other ribotype 027 isolates; however, in this case, *C*. *difficile* faecal shedding was used as an indication of colonization. It is currently unknown whether colonization of the hamster gut is needed to cause pathology and disease (or mortality). Using the early time points in infection we would like to be able to investigate possible colonization factors of *C*. *difficile* and compare mutants defective in such factors.

Histology samples at 24 h post-infection show some pathology mainly to the epithelial brush border even though very little toxin activity could be measured using a Vero cell toxicity assay. Rolfe & Song (1993) showed that carbohydrates from brush border membranes of hamster ileum can bind toxin A. It is hypothesized that TcdA acts to disrupt the epithelial layer allowing access of TcdB to its receptor on the basolateral membrane (Stubbe *et al.*, 2000). Potentially this could be reflected *in vivo* by variation in the temporal expression of these two genes (this is currently being investigated). Interestingly gut damage at 24 h (presumably due to toxin production) occurred at the same time as sporulation. Whether *in vivo* toxin production and sporulation are linked is unclear; however, Underwood *et al.* (2009) reported that disruption of the sporulation initiation pathway (via *spo0A* inactivation) also resulted in decreased toxin production in *C*. *difficile* strain 630.

Most pathological damage was seen at 36 h post-infection and onwards, and this was associated with increased bacterial numbers and greater toxin production. *C*. *difficile* harbours a putative quorum-sensing pathway, akin to *Vibrio harveyi*, that may control toxin production *in vivo* through cell-to-cell signalling; although, Carter *et al.* (2005) showed that exogenous AI-2 did not affect toxin production. Studies have shown that intestinal damage occurs mostly through the actions of TcdA/B; however, *C*. *difficile* R20291 also produces the binary toxin CDT. CDT has been shown to cause fluid accumulation in a rabbit ileal loop model but not to cause disease in hamsters (Geric *et al.*, 2006). Through the induction of microtubule-based protrusions, CDT can enhance the adherence of *C*. *difficile* bacteria to host cells (Schwan *et al.*, 2009). The activity of CDT was not measured in this study and could play an adjunctive role to TcdA/B.

CDIs exert a substantial burden on human health and economy, and there is a pressing need for vaccines and/or treatment. As infection of the hamster with *C*. *difficile* mirrors many symptoms of CDI in humans this model is ideally placed to test such interventions. *C*. *difficile* host–pathogen kinetics are essential to determine the impact of such interventions, here we detail the infection process of a clinically relevant *C*. *difficile* strain, R20291. In conclusion, the epidemic *C*. *difficile* strain R20291 caused rapid death in the hamster model of infection, where high bacterial (including spores) and toxin levels caused significant tissue damage in the caecum and colon.

## References

[r1] Bartlett J. G. Onderdonk A. B. Cisneros R. L. Kasper D. L. **(**1977**).** Clindamycin-associated colitis due to a toxin-producing species of *Clostridium* in hamsters. J Infect Dis 136, 701–705 10.1093/infdis/136.5.701 915343

[r2] Carter G. P. Purdy D. Williams P. Minton N. P. **(**2005**).** Quorum sensing in *Clostridium difficile*: analysis of a LuxS-type signalling system. J Med Microbiol 54, 119–127 10.1099/jmm.0.45817-0 15673504

[r3] Cartman S. T. Heap J. T. Kuehne S. A. Cockayne A. Minton N. P. **(**2010**).** The emergence of ‘hypervirulence’ in *Clostridium difficile*. Int J Med Microbiol 300, 387–395 10.1016/j.ijmm.2010.04.008 20547099

[r4] Geric B. Carman R. J. Rupnik M. Genheimer C. W. Sambol S. P. Lyerly D. M. Gerding D. N. Johnson S. **(**2006**).** Binary toxin-producing, large clostridial toxin-negative *Clostridium difficile* strains are enterotoxic but do not cause disease in hamsters. J Infect Dis 193, 1143–1150 10.1086/501368 16544255

[r5] Goulding D. Thompson H. Emerson J. Fairweather N. F. Dougan G. Douce G. R. **(**2009**).** Distinctive profiles of infection and pathology in hamsters infected with *Clostridium difficile* strains 630 and B1. Infect Immun 77, 5478–5485 10.1128/IAI.00551-09 19752031 PMC2786451

[r6] HC **(**2006**).** Investigation into Outbreaks of Clostridium difficile at Stoke Mandeville Hospital, Buckinghamshire Hospitals NHS Trust, http://www.cqc.org.uk/_db/_documents/Stoke_Mandeville.pdf, ISBN: 1-84562-103-4. London: Healthcare Commission

[r7] Larson H. E. Borriello S. P. **(**1990**).** Quantitative study of antibiotic-induced susceptibility to *Clostridium difficile* enterocecitis in hamsters. Antimicrob Agents Chemother 34, 1348–1353 2386366 10.1128/aac.34.7.1348 PMC175979

[r8] Lawley T. D. Croucher N. J. Yu L. Clare S. Sebaihia M. Goulding D. Pickard D. J. Parkhill J. Choudhary J. Dougan G. **(**2009**).** Proteomic and genomic characterization of highly infectious *Clostridium difficile* 630 spores. J Bacteriol 191, 5377–5386 10.1128/JB.00597-09 19542279 PMC2725610

[r9] Marsh J. W. O’Leary M. M. Shutt K. A. Pasculle A. W. Johnson S. Gerding D. N. Muto C. A. Harrison L. H. **(**2006**).** Multilocus variable-number tandem-repeat analysis for investigation of *Clostridium difficile* transmission in hospitals. J Clin Microbiol 44, 2558–2566 10.1128/JCM.02364-05 16825380 PMC1489528

[r10] Razaq N. Sambol S. Nagaro K. Zukowski W. Cheknis A. Johnson S. Gerding D. N. **(**2007**).** Infection of hamsters with historical and epidemic BI types of *Clostridium difficile*. J Infect Dis 196, 1813–1819 10.1086/523106 18190262

[r11] Rolfe R. D. Song W. **(**1993**).** Purification of a functional receptor for *Clostridium difficile* toxin A from intestinal brush border membranes of infant hamsters. Clin Infect Dis 16 Suppl. 4 S219–S227 10.1093/clinids/16.Supplement_4.S219 8324123

[r12] Rupnik M. Avesani V. Janc M. Von Eichel-Streiber C. Delmée M. **(**1998**).** A novel toxinotyping scheme and correlation of toxinotypes with serogroups of *Clostridium difficile* isolates. J Clin Microbiol 36, 2240–2247 9665999 10.1128/jcm.36.8.2240-2247.1998 PMC105025

[r13] Sambol S. P. Tang J. K. Merrigan M. M. Johnson S. Gerding D. N. **(**2001**).** Infection of hamsters with epidemiologically important strains of *Clostridium difficile*. J Infect Dis 183, 1760–1766 10.1086/320736 11372028

[r14] Schwan C. Stecher B. Tzivelekidis T. Van Ham M. Rohde M. Hardt W. D. Wehland J. Aktories K. **(**2009**).** *Clostridium difficile* toxin CDT induces formation of microtubule-based protrusions and increases adherence of bacteria. PLoS Pathog 5, e1000626 10.1371/journal.ppat.1000626 19834554 PMC2757728

[r15] Stabler R. A. He M. Dawson L. Martin M. Valiente E. Corton C. Lawley T. D. Sebaihia M. Quail M. A. **(**2009**).** Comparative genome and phenotypic analysis of *Clostridium difficile* 027 strains provides insight into the evolution of a hypervirulent bacterium. Genome Biol 10, R102 10.1186/gb-2009-10-9-r102 19781061 PMC2768977

[r16] Stubbe H. Berdoz J. Kraehenbuhl J. P. Corthésy B. **(**2000**).** Polymeric IgA is superior to monomeric IgA and IgG carrying the same variable domain in preventing *Clostridium difficile* toxin A damaging of T84 monolayers. J Immunol 164, 1952–1960 10657645 10.4049/jimmunol.164.4.1952

[r17] Underwood S. Guan S. Vijayasubhash V. Baines S. D. Graham L. Lewis R. J. Wilcox M. H. Stephenson K. **(**2009**).** Characterization of the sporulation initiation pathway of *Clostridium difficile* and its role in toxin production. J Bacteriol 191, 7296–7305 10.1128/JB.00882-09 19783633 PMC2786572

[r18] Warny M. Pepin J. Fang A. Killgore G. Thompson A. Brazier J. Frost E. McDonald L. C. **(**2005**).** Toxin production by an emerging strain of *Clostridium difficile* associated with outbreaks of severe disease in North America and Europe. Lancet 366, 1079–1084 10.1016/S0140-6736(05)67420-X 16182895

[r19] Wilcox M. H. Cunniffe J. G. Trundle C. Redpath C. **(**1996**).** Financial burden of hospital-acquired *Clostridium difficile* infection. J Hosp Infect 34, 23–30 10.1016/S0195-6701(96)90122-X 8880547

